# Microembolic signals and strategy to prevent gas embolism during extracorporeal membrane oxygenation

**DOI:** 10.1186/1749-8090-5-5

**Published:** 2010-02-04

**Authors:** Paolo Zanatta, Alessandro Forti, Enrico Bosco, Loris Salvador, Maurizio Borsato, Fabrizio Baldanzi, Carolina Longo, Carlo Sorbara, Pierluigi longatti, Carlo Valfrè

**Affiliations:** 1 Anestesia and Intensive Care Department, Treviso Regional Hospital, Italy; 2 Cardiovascular Disease Department, Treviso Regional Hospital, Italy; 3 Regional Project for the Reduction of Neurodysfunction after Cardiac Surgery and Neurosurgery, Improvement of Multimodality Neuromonitoring, Regione Veneto, Italy; 4 Neurosurgery Department, Treviso Regional Hospital, University of Padova, Italy

## Abstract

**Background:**

Extracorporeal membrane oxygenation (ECMO) supplies systemic blood perfusion and gas exchange in patients with cardiopulmonary failure. The current literature lacks of papers reporting the possible risks of microembolism among the complications of this treatment.

In this study we present our preliminary experience on brain blood flow velocity and emboli detection through the transcranial Doppler monitoring during ECMO.

**Methods:**

Six patients suffering of heart failure, four after cardiac surgery and two after cardiopulmonary resuscitation were treated with ECMO and submitted to transcranial doppler monitoring to accomplish the neurophysiological evaluation for coma.

Four patients had a full extracorporeal flow supply while in the remaining two patients the support was maintained 50% in respect to normal demand.

All patients had a bilateral transcranial brain blood flow monitoring for 15 minutes during the first clinical evaluation.

**Results:**

Microembolic signals were detected only in patients with the full extracorporeal blood flow supply due to air embolism.

**Conclusions:**

We established that the microembolic load depends on gas embolism from the central venous lines and on the level of blood flow assistance.

The gas microemboli that enter in the blood circulation and in the extracorporeal circuits are not removed by the membrane oxygenator filter.

Maximum care is required in drugs and fluid infusion of this kind of patients as a possible source of microemboli. This harmful phenomenon may be overcome adding an air filter device to the intravenous catheters.

## Background

ECMO is a well consolidated method of treatment for patients with heart failure after cardiac surgery besides intraortic balloon pump and pharmacological therapy [1]. Mortality and morbidity of this life saving procedure remain still high [2]. The more frequent complications are bleeding, renal failure, lower limb ischemia and brain iniury like cerebral haemorrhage and oedema.

Our preliminary experience in monitoring ECMO patients sustains the possible role of systemic microembolism in increasing patient morbidity.

## Methods

We investigated six consecutive patients treated with ECMO; four patients were submitted to the extracorporeal treatment because of refractory postcardiotomy cardiogenic shock while the others two patients immediately after a cardiopulmonary resuscitation for cardiac arrest (table 1). All patients were submitted to a transcranial doppler to complete the neurophysiological evaluation for coma.

**Table 1:** Patient data

	ECMO ASSISTANCE 50%		ECMO ASSISTANCE 100%			
**Patient (n)**	1	2	3	4	5	6

**Age (years)**	65	70	73	23	58	48

**Body Surface Area (m^2^)**	2,5	1,84	1,91	1,7	2	1,65

**Gender**	M	M	M	F	M	F

**Type of event**	CABG	CABG	POST CPR	POST CPR	CABG	PORT ACCESS

**Reason for ECMO**	Left ventricular failure	Left ventricular failure	Biventricular failure	Biventricular failure	Left ventricular failure	Biventricular failure

**Length of ECMO (days)**	6	12	4	11	4	2

**ECMO flow (l/min)**	3,1	2,2	4,6-5,3	4,1	4,9	3,96

**Weaned from ECMO**	yes	yes	no	yes	no	yes

**ICU Recovery Time (days)**	154	32	4	96	4	5

Demographics of patients

The neurosonology evaluation consists of the standard examination of the brain blood flow velocity of intracranial arteries (first step) and fifteen minutes monitoring with emboli count and differentiation by ultrasound probes placed on the middle cerebral arteries through the transtemporal windows (second step) with Doppler box - DWL.

The ECMO perfusion system consists of a Jostra- Maquet coated minicircuit, a Levitronix centrifugal pump and a Quadrox D oxygenator. Venous drainage was performed with a 21 F Byomedicus Carmeda multistage cannula with the distal portion proximal to the right atrium. Arterial perfusion was maintain with a 17 F Biomedicus Carmeda cannula. Both venous and arterial femoral cannula were inserted percutaneously. A heat exchanger was used to maintain a nasopharyngeal temperature between 34-35°C. All patients were sedated to reduce shivering and excess of oxygen consumption.

The patients received a perfusion assistance according to the heart function monitored by transesophageal echocardiogram and metabolic parameters from blood sampling. Air/oxygen flow was set to maintain a partial oxygen pressure of 150-200 mmHg and a carbon dioxide of 35-40 mmHg. Continuously intravenous infusion of heparin was administered to obtain an activated clotting time between 180-200 seconds.

All patients were assisted with intraortic blood pulsation and with continuous renal replacement therapy.

## Results

The standard TCD evaluation showed intracranial brain blood flow velocity in normal range in all patients except the patient 4 in which we found a high brain blood flow velocity because of a non convulsive status epilecticus detected with a subsequent electroencephalogram.

During the neurosonology monitoring we noted that only the patients treated with a complete blood flow supply suffered of brain microembolism. We detected a direct correlation between air embolism from the central infusion lines and brain microembolic signals (MES) (additional file 1). In patient 5 we registered a shower effect related to gas embolism along the introducer jugular catheter (additional file 2). We realize that gas embolisms were triggered by air microbubbles in the continuous infusion pumps, or in the crystalloid fast infusion solutions or sometimes from unlocked catheters taps.

**Figure 1:**
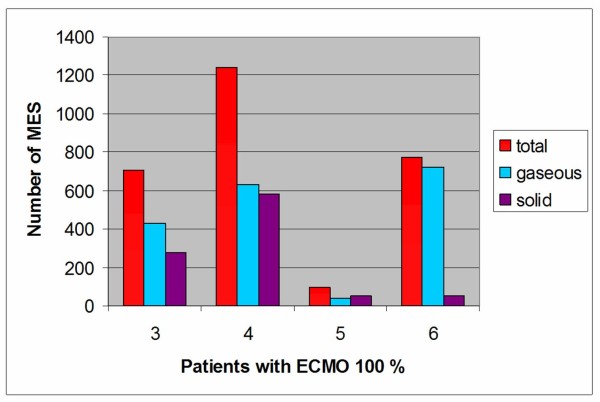
**Graphic 1**. Emboli detection and differentiation during 15 minutes TCD recording in patient 3, 4, 5, 6 submitted to ECMO 100%.

The figure 1 show the emboli count and differentiation during a 15 minutes transcranial doppler (TCD) monitoring in patients treated with ECMO 100%.

The two patients treated with 50% blood flow supply did not show any MES during the brain monitoring. Moreover the bubbles tests performed in these two patients did not show any microembolism during the ECMO perfusion (additional file 3).

**Figure 2:**
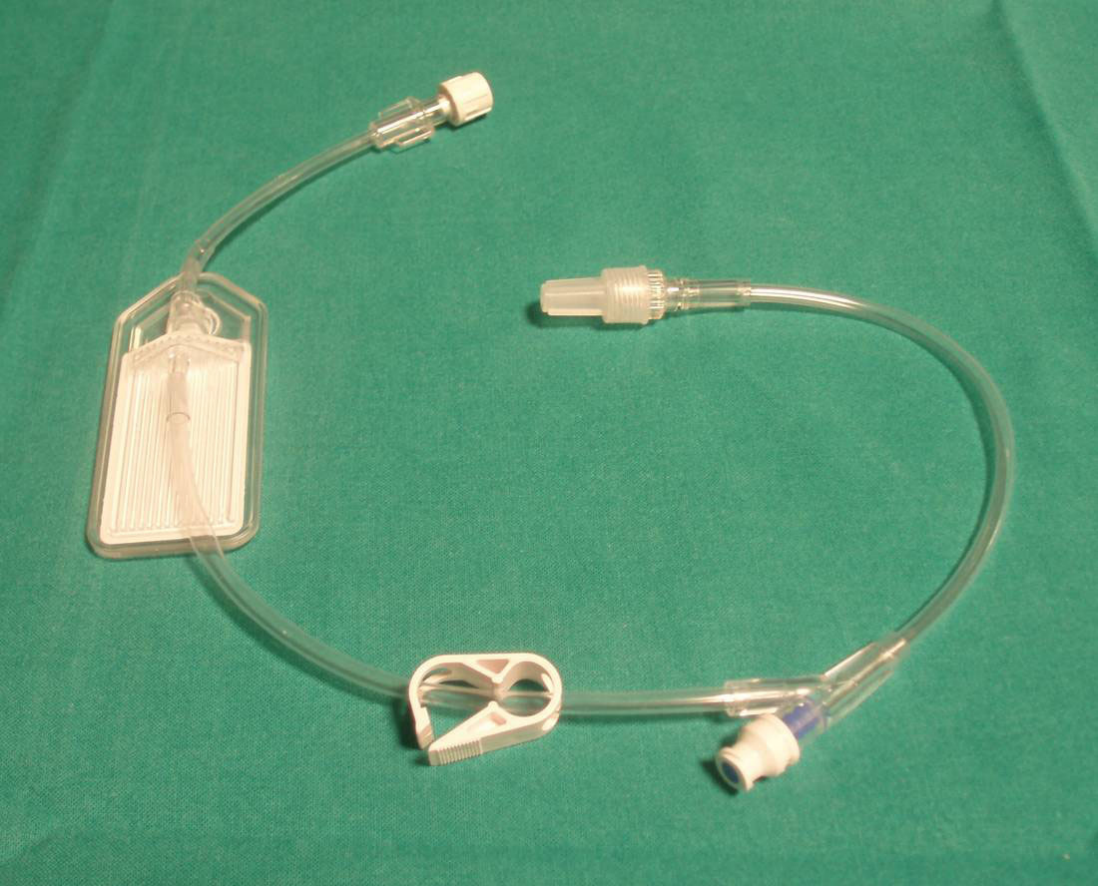
**Air filter device (SmartSite Extension Set- Cardinal Health)**. The device is placed between the intravenous catheter and the infusion lines.

To reduced the microembolic injury from venous gas embolism we tested an air filter device attached in sequence to the intravenous catheters that resulted very efficient in removing air bubbles from crystalloid solution (SmartSite Extension Set - CardinalHealth, Figure 2), (additional file 4).

Patient 3 and 5 treated with the ECMO 100% assistance died because of a multiorgan failure while, the others four patients were successfully weaned from extracorporeal circulation. Only patient 4 had maior neurologic disorders caracterized by a minimal conscious state and a spastic cerebral palsy. The others 3 patient (patient number 1, 2 and 6) had a normal neurological examination. It wasn't possible to perform neurocognitive evaluations in these three patients during the post intensive care recovery time.

## Discussion

Microemboli produced during extracorporeal circulation are a recognised cause of increasing morbidity and mortality in cardiac surgery procedures [3,4]. We agree that TCD can be helpful in assessing and monitoring the quality of extracorporeal perfusion in regard of blood flow velocity and emboli detection, allowing the best perfusion strategy, not only during cardiopulmonary bypass, but also during ECMO.

In literature there aren't papers about the brain emboli detection monitoring with TCD during ECMO. There are few reports about brain emboli monitoring with TCD on patients treated with left ventricular assist device (LVAD) [5,6]. One paper reports that in Novacor LVAD patients the amount of MES is directly correlated with clinical thromboembolism [7]; the pathogenesis of microembolization in LVAD-patients seem to be still unknown.

Our experience sustains the hypothesis that patient treated with high blood flow ECMO can be at high risk for brain and systemic embolization from venous gas embolism. Mini extracorporeal circuits and the membrane oxygenator filters seem to be not efficient enough to remove microemboli. We believe that the 50% ECMO assistance is not associated with microembolic signals because the microemboli enter more easily into the pulmonary circulation. The bubbles test performed with a mixture of 1 ml of air and 9 ml of crystalloid solution, injected into a central venous catheter sustains our hypothesis.

In the last years Doppler technology has made possible to differentiate not only gaseous but also solid microemboli [8]. The solid component of the embolic load that we detected is probably related to platelets aggregation on gas microbubbles, as documented by others papers [9]. The different proportion of solid emboli in four patients treated with ECMO 100%, could be explained with a difference in the coagulation status of the patients, despite activated clotting time was in the same range. Gaseous and solid microemboli can produce tissue ischemia and subsequent tissue damage through the obstruction of the microcirculation. On account of this we hypothesize that the pharmacological anticoagulation requested to maintain the extracorporeal circulation may be a risk factor of subsequent brain haemorrhage [2].

Our study supports the possible role of microembolization in worsening the patients outcome since the total embolic load can be enormous during several days of ECMO assistance.

Moreover in two of our patients the emboli count is underestimated because they had showers of MES (patient 5 and 6) not countable by the software for the high numbers of microbubbles that occur at once. Some authors recently proposed a radio-frequency based TCD analysis to overcome this limitation and better correlate the neurologic outcome to cerebral embolic load [10].

To reduce the probability of microembolization on ECMO we tested an air filter device (0.2 micron), high efficient in removing air bubbles from venous infusion lines (Figure 2 and additional file 4). The use of this device has made possible to erase microemboli during crystalloid solution infusion. This investigation is still open during fast infusion of fresh frozen plasma and red packed cells, because a bigger filter capacity is required. Moreover there is evidence, in patients with mechanical heart valves, that gas microbubbles may be reduce by administering 100% oxygen through the mechanism of blood de-nitrogenation [11]. This protective effect could be utilize to reduce number and size of air microbubbles also during extracorporeal perfusion.

## Conclusions

Patients treated with high flow ECMO assistance can suffer from microemboli because venous gas embolism. The extracorporeal perfusion system is not enough efficient to remove air microbubbles. During this procedure maximum care is required on setting and managing the infusion lines. The interposition of an air filter device between the intravenous catheter and the infusion lines can prevent air embolization.

Although more studies are required to verify the impact of brain and systemic microembolism during ECMO on patients outcome, we decided that this kind of investigation must not necessarily be submitted to our ethical committee, because the gas embolism can be easily solved with a simple device.

Finally TCD monitoring proved a great utility in verifying the quality of perfusion during ECMO, both for bilateral brain blood flow velocity and emboli detection.

## Abbreviations

(ECMO): Extracorporeal membrane oxygenation; (MES): Microembolic signals; (TCD): Transcranial Doppler; (LVAD): left ventricular assist device

## Competing interests

The authors declare that they have no competing interests.

## Authors' contributions

PZ conceived the work, carried out the study, collected and analyzed the data and wrote the article. AF, EB participated in the design of the study analized the data and helped to write the article, LS analyzed the data and helped to write the article. FB and CL collected the TCD data, MB collected the perfusion data. CV, CS, PLL analysed and review the article and have given final approval of the version to be published. All authors read and approved the final manuscript.

## Consent

Written informed consent was obtained from the patient for publication of this case report and accompanying images. A copy of the written consent is available for review by the Editor-in-Chief of this journal.

## Supplementary Material

Additional file 1 **Movie patient 3**. TCD microembolic signals recording from venous gas embolism. Click here for file

Additional file 2 **Movie patient 5**. TCD microembolic signal recordings from venous gas embolism. A double sample TCD recording on the right and left medial cerebral arteries is made. Note the high median brain blood flow velocity related to the effect of intraortic balloon pulsation. Note the shower effect at the start of MES recording. Click here for file

Additional file 3 **Movie patient 1**. TCD monitoring during a bubble test in ECMO 50% supply. A double sample TCD recording on the right and left medial cerebral arteries is made. Note also the M-mode and median brain blood flow velocity monitoring. Click here for file

Additional file 4 **Movie air filter**. Efficiency of the SmartSite Extension Set in removing air from crystalloid solution. Methylene blu is added to the solution to better visualize the dearing. Click here for file
